# Robust Adaptive Control Strategy for a Bidirectional DC-DC Converter Based on Extremum Seeking and Sliding Mode Control

**DOI:** 10.3390/s23010457

**Published:** 2023-01-01

**Authors:** Hoai-An Trinh, Duc Giap Nguyen, Van-Du Phan, Tan-Quoc Duong, Hoai-Vu-Anh Truong, Sung-Jin Choi, Kyoung Kwan Ahn

**Affiliations:** 1 Department of Mechanical Engineering, University of Ulsan, Ulsan 44610, Republic of Korea; 2 School of Mechanical Engineering, Kyungpook National University, Daegu 41566, Republic of Korea; 3 Department of Electrical, Electronic and Computer Engineering, University of Ulsan, Ulsan 44610, Republic of Korea; 4 Department of Mechanical Engineering, Pohang University of Science and Technology (POSTECH), Gyeongbuk 37673, Republic of Korea

**Keywords:** bidirectional DC-DC converter, voltage regulation, continuous sliding mode control, extended state observer, extremum seeking

## Abstract

This paper presents a new control strategy that combines classical control and an optimization scheme to regulate the output voltage of the bidirectional converter under the presence of matched and mismatched disturbances. In detail, a control-oriented modeling method is presented first to capture the system dynamics in a common canonical form, allowing different disturbances to be considered. To estimate and compensate for unknown disturbances, an extended state observer (ESO)-based continuous sliding mode control is then proposed, which can guarantee high tracking precision, fast disturbance rejection, and chattering reduction. Next, an extremum seeking (ES)-based adaptive scheme is introduced to ensure system robustness as well as optimal control effort under different working scenarios. Finally, comparative simulations with classical proportional-integral-derivative (PID) control and constant switching gains are conducted to verify the effectiveness of the proposed adaptive control methodology through three case studies of load resistance variations, buck/boost mode switching, and input voltage variation.

## 1. Introduction

Bidirectional DC-DC converters play an important role as a regulator to achieve the desired output of voltage or current and stabilize the DC bus in numerous applications such as hybrid microgrid systems [1,2], hybrid electric vehicles [3], fuel cell hybrid systems [4], and photovoltaic or wind energy systems [5,6]. In the hybrid system, the bidirectional DC-DC converter (BDC) is used to transform power between energy storage sources (battery, supercapacitor,…) and DC bus in two directions with a boost mode for delivering energy to load power demand and a buck mode for charging the regenerative energy back to storage sources. It can be said that the system qualification is significantly affected by this converter’s characteristics. Despite being widely commercialized and utilized in a variety of systems, the BDC control has been receiving constant attention due to existing problems of voltage ripple, output tracking performance subject to time-varying system parametric uncertainties, and matched and mismatched disturbances. Consequently, developing a control strategy that can address these regards and enhance system performance still remains challenging.

Concerning the control of DC-DC converters, several exciting methodologies have been suggested for disturbances rejection, sustaining the output voltage regulation, and achieving system performance such as backstepping control [7,8], feedback control [9,10], droop control [11,12], coefficient diagram method [13], sliding mode control (SMC) [14,15]. Among these methods, the SMC is known as an advanced technique that has attracted a lot of interest due to its significant benefits in terms of robustness against perturbations and parameter variations, ease of implementation, and reference for many industrial applications. Several works using this technique for voltage regulation were reported [16,17,18,19,20,21]. Qi et al. [16] presented a new structure of a DC-DC boost converter combined with a conventional sliding mode controller to improve the DC voltage gain and reduce voltage stress on the power switch. Although the output voltage is about 12% by the experimental tests, this proposed approach can be used to decrease the circuit’s dynamic losses with a low-duty cycle and give high efficiencies. In order to regulate the output voltage of the DC-DC boost converter, a cascade method of proportional-integral (PI) and SMC [17] was proposed based on the locus of a perturbed relay system. This strategy precisely determined the frequency and amplitude of the self-sustaining ripple, as well as the equivalent gain of the relay function, which could improve the system stability and performance under different experimental tests. Meanwhile, an improved super-twisting SMC method [18] was proposed to mitigate the chattering effect and improve the response speed of the bus voltage for a bidirectional DC-DC converter. The obtained simulation results show that the proposed strategy can reduce system overshoot by 6.8% and increase response speed by 38% in comparison to the traditional super-twisting SMC method. In [19], a second-order SMC was designed to eliminate parameter uncertainties and guarantee the output voltage of the DC-DC buck converter in fast step-load and start-up transient responses without using the current sensing or an integral term in the control loop. The high-order SMC [20] was also applied for the DC-DC boost converter of a photovoltaic system to regulate the output voltage and improve performance. In this work, the integral component was used to ensure that the system draws to the sliding surface at any point of operation, thereby eliminating chattering and steady-state error and improving system performance. Regarding mismatched disturbances, Wang et al. [21] proposed a discretized fast terminal sliding mode control for integrating disturbance compensations. The acquired results convinced that the suggested solution achieved accurate voltage tracking and faster transient response in various operating conditions. Conventionally, fixed controller gains are designed regarding the supposedly predetermined upper bound of the perturbation, matched uncertainty, or heuristic method to get the best performance. However, the drawback of this former exposes in large gains are adopted for small perturbations and vice versa. Moreover, the discontinuous term in the switching control law is another problem that causes the chattering phenomenon and potentially degrades the system qualification. To deal with this regard, an adaptive law is involved to overcome this problem and satisfy the time-varying perturbation [22]. In [23], an adaptive strategy was constructed by using a normalized output error-based SMC. This proposed control scheme offered more flexibility in tuning the controller gains, significantly improving transient responses. Furthermore, an ES method is known as a useful optimization tool, besides widely conventional techniques like genetic algorithm or particle swam optimization, to adjust the control gains to the optimal value for system qualification enhancement [24]. However, the application of ES in electronic systems is still quite limited. For these concerns, developing an adaptive continuous sliding mode control (CSMC) based on ES is an effective approach to address the effect of time-varying matched uncertainty while satisfying the requirements of the DC-DC converter operation.

As the SMC characteristics, this algorithm is only robust against perturbation but sensitive to a mismatched disturbance, which is inevitably present in any practical application. Thus, the observer technique is commonly considered to suppress the influence of these mismatched elements. In Reference [25], a disturbance observer (DO) was implemented to estimate the unknown time-varying mismatched disturbances induced by load variation and thus stabilize the DC bus voltage. Another DO [26] was also proposed to determine the unknown disturbance, which was then eliminated by linearizing feedback control to guarantee asymptotic regulation and improve the accuracy of tracking voltage. Generally, this approach requires certain information on system dynamics when being applied which may restrict the implementation in the case of not well-identified models. In contrast, the ESO possesses the property of simplicity and ease of implementation with canonical form and less system information required in comparison to the former. The effectiveness of using this technique has been examined regarding published works in observing the system states (input/output voltage/current) and realizing disturbances in the DC-DC converters for practical applications [27,28]. The experimental results of these studies confirmed the reliability of the suggested technique, in which the ESO was a preferable method in mismatched disturbance suppression with extreme reliability. Therefore, the combination of ES-based CSMC and ESO has the potential to expand interesting research topics for high-precision tracking control of DC-DC converters, as well as expansions into related fields of study such as energy management control or optimization-control techniques.

Motivated by the aforementioned shortcomings, this paper focuses on developing an ES-based adaptive CSMC with disturbance rejection enhanced by using an ESO to improve the tracking performance of a bidirectional DC-DC converter. The main contributions of this study can be summarized in the following points.

- The objectives of tracking accuracy, disturbance suppression, and chattering alleviation are overall achieved by the proposed ESO-based CSMC.
- An ES-based adaptive scheme particularly designed for CSMC is proposed to adjust the switching gain, which can reduce the voltage ripple and the magnitude of the peaking phenomenon.
- The stability of the proposed strategy for the whole system is theoretically proven by the Lyapunov approach in the influence of the matched and mismatched disturbances.
- The validation is performed to verify the effectiveness and reliability of the proposed strategy under three case studies of load resistance variation, buck/boost mode switching, and input voltage variation.

The rest of this paper is organized as follows. Section 2 presents the nonlinear model of the bidirectional DC-DC converter. Section 3 introduces the proposed control algorithm including mismatched disturbance estimation by ESO, voltage tracking and disturbance rejection by CSMC, switching parameter adaptation by ES, and stability analysis. Furthermore, the system setup and simulation results are given in Section 4. Ultimately, a conclusion is given in Section 5.

## 2. Nonlinear Model of Bidirectional DC-DC Converter

### 2.1. Generalities of the Bidirectional Converter Circuit

**Figure 1:**
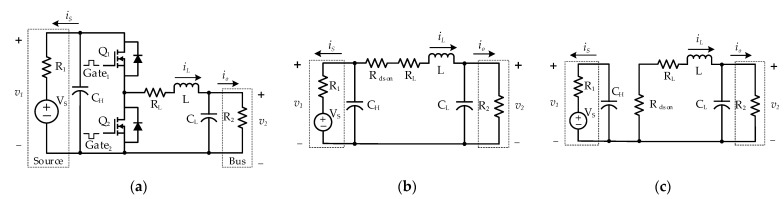
Schematic diagram of a bidirectional DC-DC converter. (**a**) The general structure of bidirectional DC-DC converter. (**b**,**c**) Equivalent circuit diagram of State 1 and State 2, respectively.

Consider a typical bidirectional DC-DC converter and equivalent circuit of the two states whose schematic diagrams are shown in Figure 1.

In this configuration, a voltage source $V_{S}$ and a DC bus are respectively located on the high-voltage side and low-voltage side of the converter. $v_{1}$ and $v_{2}$ are the voltage at the high and low sides of the converter, respectively. $R_{1}$ denotes the internal resistance of the voltage source, whereas, in the simplest case, $R_{2}$ is considered to be a load resistance. The inductor L is accompanied by a parasitic resistance, $R_{L}$. $R_{\mathrm{dson}}$ indicates the MOSFET turn-on resistance. Capacitor $C_{H}$ and $C_{L}$ represent the input and output capacitor of the converter, respectively. $Q_{1}$ and $Q_{2}$ are two active switches that are directly controlled by two complementary gating signals ${\mathrm{Gate}}_{1}$ and ${\mathrm{Gate}}_{2}$, separately. As a result, the inductor current $i_{L}$, the input current $i_{S}$, and the output current $i_{0}$ can fundamentally flow in both directions. Because of the complementary control signal, no matter what mode (buck or boost) the bidirectional converter is in, there are only two states, namely State 1 ($Q_{1}$ ON–$Q_{2}$ OFF) and State 2 ($Q_{1}$ OFF–$Q_{2}$ ON). These states are described by equivalent circuits as shown in Figure 1b,c. The governing equation sets for these states can be expressed as:

(1) $$\mathrm{State}\;1:\;\;\left\{ \begin{array}{l} L{\dot{i}}_{L}=-i_{L}R_{\mathrm{eq}}+v_{1}-v_{2}\\ C_{H}{\dot{v}}_{1}=-i_{L}-\frac{1}{R_{1}}v_{1}+\frac{V_{S}}{R_{1}}\\ C_{L}{\dot{v}}_{2}=i_{L}-\frac{1}{R_{2}}v_{2} \end{array}\right.$$

(2) $$\mathrm{State}\;2:\;\;\left\{ \begin{array}{l} L{\dot{i}}_{L}=-i_{L}R_{\mathrm{eq}}-v_{2}\\ C_{H}{\dot{v}}_{1}=-\frac{1}{R_{1}}v_{1}+\frac{V_{S}}{R_{1}}\\ C_{L}{\dot{v}}_{2}=i_{L}-\frac{1}{R_{2}}v_{2} \end{array}\right.$$

where $R_{\mathrm{eq}}=\;R_{\mathrm{dson}}+R_{L}$.

Since the two active switches $Q_{1}$ and $Q_{2}$ are controlled complementarily, there should be a single controller to operate the bidirectional converter. Hence, two governing equation sets (1) and (2) could be fused by introducing the duty cycle variable μ∈[0,1]. This action results in the following average modeling equation set.

(3) $$\left\{ \begin{array}{l} L{\dot{i}}_{L}=-i_{L}R_{\mathrm{eq}}+\mu v_{1}-v_{2}\\ C_{H}{\dot{v}}_{1}=-\mu i_{L}-\frac{1}{R_{1}}v_{1}+\frac{V_{S}}{R_{1}}\\ C_{L}{\dot{v}}_{2}=i_{L}-\frac{1}{R_{2}}v_{2} \end{array}\right.$$

However, it is expected that in practice the target system might experience several disturbances, namely resistive load variations, unmodeled terms, or parameter uncertainties. Consequently, the average modeling equation set (3) can be modified as:

(4) $$\left\{ \begin{array}{l} L{\dot{i}}_{L}=-i_{L}R_{\mathrm{eq}}+\left(\bar{\mu }+\Delta \mu \right)v_{1}-v_{2}\\ C_{H}{\dot{v}}_{1}=-\left(\bar{\mu }+\Delta \mu \right)i_{L}-\frac{1}{R_{1}}v_{1}+\frac{V_{S}}{R_{1}}\\ C_{L}{\dot{v}}_{2}=i_{L}-\frac{1}{{\;\bar{R}}_{2}}v_{2}-\left(\frac{1}{R_{2}}-\frac{1}{{\;\bar{R}}_{2}}\right)v_{2} \end{array}\right.$$

where $\bar{\mu }$ and ${\;\bar{R}}_{2}$ denote the nominal duty cycle and nominal load resistance, respectively, Δμ represents the duty cycle perturbation, and $R_{2}$ is the actual load resistance.

### 2.2. Control-Oriented Modeling

Let $x_{1}=e={\mathrm{LC}}_{L}\left(v_{2}-V_{r}\right)$ be the first system state, where $V_{r}$ denotes the desired output voltage. The system model can be rewritten as follows.

(5) $$\begin{array}{cc} {\dot{x}}_{1} & =\dot{e}={\mathrm{LC}}_{L}{\dot{v}}_{2}=Li_{L}-\frac{L}{{\;\bar{R}}_{2}}v_{2}-L\left(\frac{1}{R_{2}}-\frac{1}{{\;\bar{R}}_{2}}\right)v_{2}\\ & =x_{2}+d_{1} \end{array}$$

where $x_{2}=Li_{L}-\left(L/{\;\bar{R}}_{2}\right)v_{2}$ represents the second system state and $d_{1}=-L\left(1/R_{2}-1/{\;\bar{R}}_{2}\right)v_{2}+\varphi_{1}$ denotes the first disturbance of the system with $\varphi_{1}$ being the first unmodeled term.

Differentiating $x_{2}$ yields:

(6) $$\begin{array}{cc} {\dot{x}}_{2} & =L{\dot{i}}_{L}-\frac{L}{{\;\bar{R}}_{2}}{\dot{v}}_{2}\\ & =-\left(\frac{R_{\mathrm{eq}}}{L}+\frac{1}{{\;\bar{R}}_{2}C_{L}}\right)x_{2}-\frac{1}{{\mathrm{LC}}_{L}}\left(\frac{R_{\mathrm{eq}}}{{\;\bar{R}}_{2}}+1\right)x_{1}+\bar{\mu }v_{1}-\left(\frac{R_{\mathrm{eq}}}{{\;\bar{R}}_{2}}+1\right)V_{r}-\frac{1}{{\;\bar{R}}_{2}C_{L}}d_{1}+\Delta \mu v_{1}+\varphi_{2}\\ & =f+u+d_{2} \end{array}$$

where

(7) $$\left\{ \begin{array}{l} f=-\left(\frac{R_{\mathrm{eq}}}{L}+\frac{1}{{\;\bar{R}}_{2}C_{L}}\right)x_{2}-\frac{1}{{\mathrm{LC}}_{L}}\left(\frac{R_{\mathrm{eq}}}{{\;\bar{R}}_{2}}+1\right)x_{1}\\ u=\bar{\mu }v_{1}-\left(\frac{R_{\mathrm{eq}}}{{\;\bar{R}}_{2}}+1\right)V_{r}\\ d_{2}=-\frac{1}{{\;\bar{R}}_{2}C_{L}}d_{1}+\Delta \mu v_{1}+\varphi_{2} \end{array}\right.$$

where $\varphi_{2}$ denotes the second unmodeled term. In the above equation set, f could be interpreted as the system dynamics, $d_{2}$ represents the second disturbance of the system, and *u* represents the virtual control input, from which the actual command duty cycle can be calculated as:

(8) $$\bar{\mu }=\frac{1}{v_{1}}\left(u+\left(\frac{R_{\mathrm{eq}}}{{\;\bar{R}}_{2}}+1\right)V_{r}\right)$$

Combining (5)–(7), one can obtain the following canonical form, which is readily available for nonlinear control algorithm design:

(9) $$\left\{ \begin{array}{l} {\dot{x}}_{1}=x_{2}+d_{1}\\ {\dot{x}}_{2}=f+u+d_{2} \end{array}\right.$$

Consequently, the problem of voltage tracking control for a bidirectional DC-DC converter now is transformed into the problem of stabilizing a second-order nonlinear system. The canonical format (9) is more suitable for nonlinear control algorithm applications than the original governing Equation (4). Moreover, in this model, different disturbances have been considered and isolated from the system dynamics such that they form only two well-known types of disturbances. Specifically, the disturbance $d_{1}$ enters the system in a different channel with the control input *u*. As a result, $d_{1}$ could be interpreted as a mismatched disturbance, whereas $d_{2}$ represents a matched one.

**Remark** **1.** *The control-oriented modeling presented in this section particularly addresses the problem of voltage regulation on the low-voltage side of the bidirectional DC-DC converter. For other tasks including voltage regulation on the high-voltage side or current control, the modeling must be tailored individually for each task. This fact, on one hand, represents a unique characteristic of the control-oriented modeling method, on the other hand, might be its limitation.*

## 3. Control Algorithm Design

**Figure 2:**
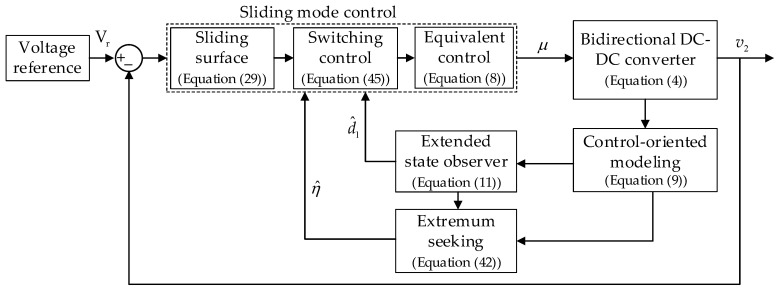
Proposed control strategy for the bidirectional DC-DC converter.

To regulate the low-voltage side $v_{2}$ of the bidirectional DC-DC converter whose dynamics model is captured in (4), a model-control scheme is proposed in Figure 2.

Figure 2 illustrates the proposed control algorithm for the converter, where different control problems are addressed as follows:

- Control-oriented modeling: transforming the average model (4) into a common canonical form with different disturbances being grouped into matched and mismatched disturbances.
- Mismatched disturbance estimation and rejection: implementing the ESO to estimate mismatched disturbances in real time and utilizing the estimation results to enhance the CSMC equivalent control signal.
- Matched disturbance suppression: leaving the matched disturbance for the robustness of a CSMC to handle.
- Adaptive scheme: switching parameter of CSMC with ES for chattering reduction, disturbance variation adaption, and optimal control signal generation.

Consider the second-order nonlinear system (9), which is subject to both matched and mismatched disturbances.

**Assumption** **1.** *Both mismatched and matched disturbances are differentiable and bounded satisfying:*

(10) $$\begin{array}{cc} \left\{ \begin{array}{l} \max\left|d_{i}(t)\right|\leq D_{i}\\ \max\left|{\dot{d}}_{i}(t)\right|\leq {\bar{D}}_{i} \end{array}\right., & i=1,2 \end{array}$$

where $D_{i}>0$, ${\bar{D}}_{i}>0$ are two unknown positive constants.

To achieve the objective of stabilizing the nonlinear system (9) in the adverse influence of disturbances, an ESO is employed to precisely estimate the mismatched disturbance in real time. Subsequently, the estimation result is utilized in a CSMC design to suppress the negative effect of disturbances, thus enhancing the disturbance rejection capability and stabilizing the performance of the control system. Meanwhile, the matched disturbance is left for the robustness of the CSMC to handle.

### 3.1. Extended State Observer for Mismatched Disturbance Estimation

Consider a linear extended state observer addressing mismatched disturbance of the following form.

(11) $$\left\{ \begin{array}{l} {\dot{\hat{x}}}_{1}=x_{2}+{\hat{d}}_{1}+\frac{\alpha_{1}}{\rho }(x_{1}-{\hat{x}}_{1})\\ {\dot{\hat{d}}}_{1}=\frac{\alpha_{2}}{\rho^{2}}(x_{1}-{\hat{x}}_{1}) \end{array}\right.$$

where ${\hat{x}}_{1}$ and ${\hat{d}}_{1}$ denote the estimation of the first state $x_{1}$ and the mismatched disturbance $d_{1}$, respectively; $\alpha_{1}$ and $\alpha_{2}$ are the observer parameters with positive constants; ρ is an arbitrarily small positive constant.

Let the estimation error of the observer (11) be:

(12) $$\begin{array}{ccc} \varepsilon ={\left[\begin{array}{cc} \varepsilon_{1} & \varepsilon_{2} \end{array}\right]}^{T}, & \varepsilon_{1}=\frac{x_{1}-{\hat{x}}_{1}}{\rho }, & \varepsilon_{2}=d_{1}-{\hat{d}}_{1} \end{array}$$

The following results can be then obtained:

(13) $$\begin{array}{cc} \rho {\dot{\varepsilon }}_{1} & ={\dot{x}}_{1}-{\dot{\hat{x}}}_{1}=\left(x_{2}+d_{1}\right)-\left[x_{2}+{\hat{d}}_{1}+\frac{\alpha_{1}}{\rho }(x_{1}-{\hat{x}}_{1})\right]\\ & =\varepsilon_{2}-\alpha_{1}\varepsilon_{1} \end{array}$$

(14) $$\begin{array}{cc} \rho {\dot{\varepsilon }}_{2} & =\rho \left({\dot{d}}_{1}-{\dot{\hat{d}}}_{1}\right)=\rho {\dot{d}}_{1}-\frac{\alpha_{2}}{\rho }\left(x_{1}-{\hat{x}}_{1}\right)\\ & =\rho {\dot{d}}_{1}-\alpha_{2}\varepsilon_{1} \end{array}$$

Subsequently, combining (13) and (14) yields the following estimation error system.

(15) $$\rho \dot{\varepsilon }=A\varepsilon +\rho B{\dot{d}}_{1}$$

where $A=\left[\begin{array}{cc} -\alpha_{1} & 1\\ -\alpha_{2} & 0 \end{array}\right]$ and $B=\left[\begin{array}{c} 0\\ 1 \end{array}\right]$.

The characteristic equation of matrix A can be expressed as follows.

(16) $$\left|\lambda I-A\right|=\left|\begin{array}{cc} \lambda +\alpha_{1} & -1\\ \alpha_{2} & \lambda \end{array}\right|=\lambda^{2}+\alpha_{1}\lambda +\alpha_{2}=0$$

From (16), if the design parameters $\alpha_{1}$ and $\alpha_{2}$ are properly selected such that the matrix A is Hurwitz, then for any given symmetric positive definite matrix Q, there exists a unique symmetric positive definite matrix P that satisfies the following Lyapunov equation [29]:

(17) $$A^{T}P+PA=-Q$$

A Lyapunov candidate is considered as

(18) $$V_{1}=\rho \varepsilon^{T}P\varepsilon$$

Define:

(19) $$\chi =\frac{\lambda_{\min}\left(Q\right)}{\lambda_{\max}\left(P\right)}$$

and suppose that the mismatched disturbance satisfies

(20) $$\left\|B{\dot{d}}_{1}\right\|\leq \frac{\chi }{2\rho }\left\|\varepsilon \right\|$$

Taking the derivative of the above Lyapunov function yields

(21) $$\begin{array}{cc} {\dot{V}}_{1} & =\rho {\dot{\varepsilon }}^{T}P\varepsilon +\rho \varepsilon^{T}P\dot{\varepsilon }={\left(A\varepsilon +\rho B{\dot{d}}_{1}\right)}^{T}P\varepsilon +\varepsilon^{T}P\left(A\varepsilon +\rho B{\dot{d}}_{1}\right)\\ & =\varepsilon^{T}A^{T}P\varepsilon +\rho {\left(B{\dot{d}}_{1}\right)}^{T}P\varepsilon +\varepsilon^{T}\mathrm{PA}\varepsilon +\varepsilon^{T}P\rho B{\dot{d}}_{1}\\ & =\varepsilon^{T}\left(A^{T}P+\mathrm{PA}\right)\varepsilon +2\rho \varepsilon^{T}\mathrm{PB}{\dot{d}}_{1} \end{array}$$

where the last equality is followed because the quantities $\rho {\left(B{\dot{d}}_{1}\right)}^{T}P\varepsilon$ and $\varepsilon^{T}P\rho B{\dot{d}}_{1}$ are scalars and thus

(22) $$\rho {\left(B{\dot{d}}_{1}\right)}^{T}P\varepsilon ={\left(\rho {\left(B{\dot{d}}_{1}\right)}^{T}P\varepsilon \right)}^{T}=\varepsilon^{T}P\rho B{\dot{d}}_{1}$$

Using (22) and the Cauchy-Schwarz inequality [30], the Lyapunov function derivative (21) becomes

(23) $$\begin{array}{cc} {\dot{V}}_{1} & =\varepsilon^{T}\left(A^{T}P+\mathrm{PA}\right)\varepsilon +2\rho \varepsilon^{T}\mathrm{PB}{\dot{d}}_{1}\\ & \leq -\varepsilon^{T}Q\varepsilon +2\rho \left\|P\varepsilon \right\|\left\|B{\dot{d}}_{1}\right\|\\ & \leq -\lambda_{\min}\left(Q\right){\left\|\varepsilon \right\|}^{2}+2\rho \lambda_{\max}\left(P\right)\left\|\varepsilon \right\|\left\|B{\dot{d}}_{1}\right\|\\ & \leq -\lambda_{\max}\left(P\right)\left\|\varepsilon \right\|\left(\frac{\lambda_{\min}\left(Q\right)}{\lambda_{\max}\left(P\right)}\left\|\varepsilon \right\|-2\rho \left\|B{\dot{d}}_{1}\right\|\right)\\ & \leq -\lambda_{\max}\left(P\right)\left\|\varepsilon \right\|\left(\chi \left\|\varepsilon \right\|-2\rho \left\|B{\dot{d}}_{1}\right\|\right) \end{array}$$

where the Rayleigh principle has been applied such that

(24) $$-\varepsilon^{T}Q\varepsilon \leq -\lambda_{\min}\left(Q\right){\left\|\varepsilon \right\|}^{2}$$

(25) $$\left\|P\varepsilon \right\|=\sqrt{\varepsilon^{T}P^{2}\varepsilon }\leq \sqrt{\lambda_{\max}\left(P^{2}\right){\left\|\varepsilon \right\|}^{2}}=\lambda_{\max}\left(P\right)\left\|\varepsilon \right\|$$

Therefore, if condition (20) is satisfied, the Lyapunov derivative (23) is always negative, and thus, the bounded stability of the observer is guaranteed. Consequently, the estimation errors will converge to a neighborhood of the origin.

**Remark** **2.** *Concerning condition (20), it is advised to select Q such that it maximizes (19) [29]. More transparently, it will certainly be beneficial to design ρ≪1 to be a sufficiently small number for higher robustness. However, a smaller ρ causes a larger peaking phenomenon if the initial condition of the first state and that of the first state estimation are different, or $x_{1}(0)\neq {\hat{x}}_{1}(0)$. In that case, ρ could be designed as follows.*

(26) $$\rho =\{\begin{array}{cc} \frac{1}{R\max\left(t^{2},r\right)} & t\leq t_{\max}\;s\\ \frac{1}{R} & \mathrm{otherwise} \end{array}$$

where *R*, *r*, and $t_{\max}$ are positive tuning numbers. The max(∗) function prevents dividing to zero.

### 3.2. Continuous Sliding Mode Control Design

Inspired by the work on matched disturbance rejection [30], this section introduces a continuous asymptotic sliding mode control to overcome the effects of both matched and mismatched disturbances and ultimately stabilize the system (9). The idea is to design a sliding mode surface so that as the sliding mode occurs, the system would follow the desired dynamic. Moreover, a sliding mode control law is designed in terms of the control function derivative. Hence, the actual control is continuous and chattering-reduced because of the integration of the high-frequency switching function.

Consider the nonlinear system (9) and let v(t) be the derivative of the control input u(t) such that

(27) $$\begin{array}{cc} u\left(t\right)=\int_{0}^{t}v\left(\tau \right)d\tau , & u\left(0\right)=0 \end{array}$$

Denote respectively the prime and auxiliary sliding mode variables of the following forms

(28) $$\sigma =x_{2}+{\hat{d}}_{1}+cx_{1}$$

(29) $$s=\dot{\sigma }+\bar{c}\sigma$$

where *c*, $\bar{c}>0$ are design positive constants, and ${\hat{d}}_{1}$ is the estimated mismatched disturbance, which is purposedly added into the sliding mode surface to suppress the influence of mismatched disturbance.

Taking the derivative of the auxiliary sliding mode variable (29) and using (9), (27), (28) yields

(30) $$\begin{array}{cc} \dot{s} & =\ddot{\sigma }+\bar{c}\dot{\sigma }\\ & =v+\dot{f}+\left(c+\bar{c}\right)f+\left(c+\bar{c}\right)u+{\ddot{\hat{d}}}_{1}+\bar{c}{\dot{\hat{d}}}_{1}+c{\dot{d}}_{1}+c\bar{c}x_{2}+c\bar{c}d_{1}+{\dot{d}}_{2}+\left(c+\bar{c}\right)d_{2} \end{array}$$

Based on (30), to compensate for the system dynamics and effects of disturbances, a high-frequency switching control v(t) can be designed as

(31) $$v=-\dot{f}-\left(c+\bar{c}\right)f-\left(c+\bar{c}\right)u-{\ddot{\hat{d}}}_{1}-\bar{c}{\dot{\hat{d}}}_{1}-c{\dot{\hat{d}}}_{1}-c\bar{c}x_{2}-c\bar{c}{\hat{d}}_{1}-\eta \mathrm{sign}\left(s\right)-k_{0}s$$

where $k_{0}>0$ is a design positive constant, η is designed later.

By substituting (31) into (30), the derivative of the auxiliary sliding mode variable now becomes

(32) $$\dot{s}=c{\dot{e}}_{d_{1}}+c\bar{c}e_{d_{1}}+{\dot{d}}_{2}+\left(c+\bar{c}\right)d_{2}-\eta \mathrm{sign}\left(s\right)-k_{0}s$$

where $e_{d_{1}}=d_{1}-{\hat{d}}_{1}$ and ${\dot{e}}_{d_{1}}={\dot{d}}_{1}-{\dot{\hat{d}}}_{1}$ denote the mismatched disturbance estimation error and its derivative, respectively.

Consider a Lyapunov candidate of the form

(33) $$V_{2}=\frac{1}{2}s^{2}$$

whose derivative can be expressed based on (32) as follows

(34) $$\begin{array}{cc} {\dot{V}}_{2} & =s\dot{s}\\ & =s\left(c{\dot{e}}_{d_{1}}+c\bar{c}e_{d_{1}}+{\dot{d}}_{2}+\left(c+\bar{c}\right)d_{2}-\eta \mathrm{sign}\left(s\right)-k_{0}s\right)\\ & \leq \left|s\right|\left(c{\bar{E}}_{d_{1}}+c\bar{c}E_{d_{1}}+{\bar{D}}_{2}+\left(c+\bar{c}\right)D_{2}-\eta \right) \end{array}$$

In (34), Assumption 1 on matched disturbance and the results of the stability analysis on mismatched disturbance estimation errors in the previous section have been utilized. Specifically, the estimation error and its derivative are implied to be bounded satisfying

(35) $$\left\{ \begin{array}{l} \max\left|e_{d_{1}}\right|\leq E_{d_{1}}\\ \max\left|{\dot{e}}_{d_{1}}\right|\leq {\bar{E}}_{d_{1}} \end{array}\right.$$

Consider the case where the switching parameter η is sufficiently selected such that

(36) $$\eta \geq \Psi =c{\bar{E}}_{d_{1}}+c\bar{c}E_{d_{1}}+{\bar{D}}_{2}+\left(c+\bar{c}\right)D_{2}+\frac{\beta }{\sqrt{2}}$$

One could obtain the following result by substituting (36) into (34) and using (33).

(37) $${\dot{V}}_{2}\leq -\beta \;\sqrt{V_{2}}$$

Separating the variables and integrating both sides of (37) over the period 0≤τ≤t yield

(38) $$\sqrt{V_{2}(t)}\leq -\frac{1}{2}\beta t+\sqrt{V_{2}(0)}$$

As a result, V(t) would reach the equilibrium in a finite time $t_{r}$ satisfying

(39) $$t_{r}\leq \frac{2\;V^{1/2}(0)}{\beta }=\frac{\sqrt{2}\left|s\left(0\right)\right|}{\beta }$$

Consequently, s(t) will converge to zero in a finite time. As the sliding mode, s(t)=0, occurs, the following results could be obtained from (29)

(40) $$\sigma \left(t\right)=\sigma \left(0\right)e^{-\bar{c}t}$$

Thus, the prime sliding mode variable σ(t) will asymptotically converge to zero. As this happens, from (28), the system dynamics now becomes

(41) $$\begin{array}{r} x_{2}+{\hat{d}}_{1}+cx_{1}=0\\ \Leftrightarrow {\dot{x}}_{1}+cx_{1}=d_{1}-{\hat{d}}_{1} \end{array}$$

Here, the asymptotic convergence of the mismatched disturbance observer on the right-hand side of the equation leads to the asymptotic convergence of the first system state $x_{1}(t)$ on the left-hand side as well. Therefore, the overall control objective is achieved.

### 3.3. Adaptive Scheme by Extremum Seeking

The switching parameter η in (36) plays a crucial role in control performance. Specifically, a large value of η would not only shorten the convergence time but also strengthen the system’s robustness against disturbances, thus ensuring tracking accuracy. Nonetheless, there is a causal relationship between the switching parameter magnitude and the chattering phenomenon observed in the control signal. In detail, a small variation in disturbance could be easily suppressed by selecting a sufficient value for the switching parameter. However, if the disturbance varies in a much wider range than expected, a consistently large switching parameter would be inefficient due to excessive chattering phenomena. In such cases, it is therefore important to adjust the switching parameter accordingly. Thus, in this section, we introduce an ES-based adaptive scheme for the switching parameter to achieve system stability, tracking accuracy, and chattering reduction as well as avoid unnecessarily large control efforts.

**Figure 3:**
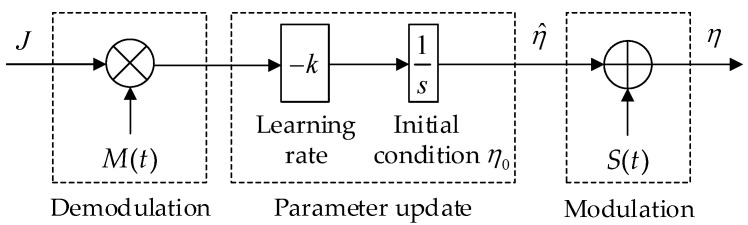
Proposed ES-based adaptive scheme for the switching parameter.

Figure 3 illustrates the proposed switching parameter optimization by ES, whose stability analysis was conclusively proved in the literature [31,32,33]. Four sequential stages include:

1. Demodulation: acquiring the gradient information by multiplying the calculated objective function *J* by another sinusoidal signal M(t)=asinωt with the same frequency ω (rad/s) as the modulation signal and *a* denotes the tuning parameter. An optional high-pass filter could be added to this stage to remove bias from the responded objective function.
2. Parameter update: updating the switching parameter $\hat{\eta }$ by integrating the demodulated signal. This stage consists of a learning rate *k*, which determines convergence speed and accuracy. An optional low-pass filter could be added to this stage to filter out high-frequency noise from the demodulated signal.
3. Modulation: perturbing the being-optimized switching parameter with a low-amplitude sinusoidal signal S(t)=bsinωt with *b* as the modulation amplitude.

An adaptive scheme for the switching parameter η could be intuitively designed based on the working principle of the sliding mode control algorithm as follows. Ideally, as the disturbance becomes large enough to influence the system, η should be sufficiently large to drive the sliding surfaces to the origin in a short amount of time. However, once the sliding mode occurs, it is unnecessary to keep η at that same level but more effective to reduce its amplitude to a level just sufficient enough to eliminate the influence of the disturbance. This action is expected to not only ensure system stability, tracking precision, and fast convergence but also reduce control effort and severity of the chattering phenomenon. From this perspective and based on the above ES scheme, the adaptive law of the switching gain is designed as

(42) $$\eta (t)=\hat{\eta }(t)+S(t)$$

(43) $$\dot{\hat{\eta }}(t)=kJM(t)$$

(44) $$J=k_{1}(k_{2}e^{2}+k_{3}s^{2})$$

where S(t)=bsinωt and M(t)=asinωt are the period perturbation signals, *k* is the learning rate, *J* describes the cost function, $k_{1}$ denotes the objective function gain, $k_{2}$ is the objective error gain, $k_{3}$ is the objective function sliding variable gain, $e=x_{1}$ is the tracking error, and *s* is the auxiliary sliding mode variable defined at (29).

**Remark** **3.** Fundamentally, the ES control algorithm might have an optional high-pass and low-pass filter in its design. However, (44) indicates that the optimal value of the objective function is $J^{*}=0$, thus a high-pass filter is not required in the scheme. Also, a low-pass filter could lead to delays in response and therefore should be refrained from use.

Based on the adaptive law (42), the high-frequency switching control v(t) (31) can be rewritten as

(45) $$v=-\dot{f}-\left(c+\bar{c}\right)f-\left(c+\bar{c}\right)u-{\ddot{\hat{d}}}_{1}-\bar{c}{\dot{\hat{d}}}_{1}-c{\dot{\hat{d}}}_{1}-c\bar{c}x_{2}-c\bar{c}{\hat{d}}_{1}-\eta \mathrm{sign}\left(s\right)-k_{0}s$$

**Remark** **4.** *ES control is a model-free optimization algorithm. Thus, its precision does not depend on the knowledge of the target system but rather lies on the objective function design and parameter selection. Together with the working principle of the sliding mode control, in (44), s should be weighed more than e, or $k_{3}>k_{2}$.*

The forcing frequency ω and the amplitudes *a*, *b* of the demodulation and modulation signals, are selected by the trial-and-error method. However, it should be ensured that the time scale of the system dynamics should be considerably smaller than that of the forcing frequency. In some cases, a time-frequency response plot of the objective function could offer a glimpse of how to select an appropriate forcing frequency. Besides, the amplitude of the demodulation signal should be much larger than that of the modulation signal, or a≫b.

### 3.4. Stability Analysis

**Theorem** **1.** *Considering the control law (31) implemented with the ESO function in (11), and the adaptive law of the switching gain (42) guarantee the stability of the closed-loop system. Then, the estimated disturbance, positive control gains, and tracking errors are bounded.*

**Proof of Theorem** **1.** The Lyapunov function can be defined as

(46) $$V=\rho \varepsilon^{T}P\varepsilon +\frac{1}{2}s^{2}+\frac{1}{2}{\tilde{\eta }}^{2}$$

We define $\tilde{\eta }≜\eta -\eta^{*}$ is the adaptive gain error with the desired optimal value $\eta^{*}$. The time derivative of the Lyapunov function (46) is expressed as follows:

(47) $$\begin{array}{cc} \dot{V} & =\rho {\dot{\varepsilon }}^{T}P\varepsilon +\rho \varepsilon^{T}P\dot{\varepsilon }+s\dot{s}+\tilde{\eta }\dot{\tilde{\eta }}\\ & =\varepsilon^{T}\left(A^{T}P+\mathrm{PA}\right)\varepsilon +2\rho \varepsilon^{T}\mathrm{PB}{\dot{d}}_{1}+s\left(c{\dot{e}}_{d_{1}}+c\bar{c}e_{d_{1}}+{\dot{d}}_{2}+\left(c+\bar{c}\right)d_{2}-\eta \mathrm{sign}\left(s\right)-k_{0}s\right)\\ & +\tilde{\eta }\left(kJM+\dot{S}\right) \end{array}$$

Substituting (23), (32), and (42) into (47), and applying Young’s inequality, one yields

(48) $$\begin{array}{cc} \dot{V} & \leq -Q{\left\|\varepsilon \right\|}^{2}+{\left(\rho \mathrm{PB}\right)}^{2}{\left\|\varepsilon \right\|}^{2}+{\dot{d}}_{1}^{2}-k_{0}s^{2}+s\left(c{\dot{e}}_{d_{1}}+c\bar{c}e_{d_{1}}+{\dot{d}}_{2}+\left(c+\bar{c}\right)d_{2}\right)+kJM\tilde{\eta }+\tilde{\eta }\dot{S}\\ & \leq -Q{\left\|\varepsilon \right\|}^{2}+{\left(\rho \mathrm{PB}\right)}^{2}{\left\|\varepsilon \right\|}^{2}+{\dot{d}}_{1}^{2}-k_{0}s^{2}+s{\dot{d}}_{2}+s\left(c+\bar{c}\right)d_{2}+sc{\dot{e}}_{d_{1}}+sc\bar{c}e_{d_{1}}+kJM\tilde{\eta }+\tilde{\eta }\dot{S}\\ & \leq -{\left\|\varepsilon \right\|}^{2}\left(Q-\rho^{2}\left(\lambda_{\max}\left(P^{T}{\mathrm{BB}}^{T}P\right)\right)\right)-\left(k_{0}-2\right)s^{2}-\frac{1}{2}\left(k^{2}-1\right){\tilde{\eta }}^{2}\\ & +\frac{1}{2}{\dot{d}}_{2}^{2}+\frac{1}{2}{\left(c+\bar{c}\right)}^{2}d_{2}^{2}+\frac{1}{2}{\dot{S}}^{2}+\frac{1}{2}{\left(JM\right)}^{2}+{\dot{d}}_{1}^{2}+\frac{1}{2}{\left(c{\bar{E}}_{d_{1}}\right)}^{2}+\frac{1}{2}{\left(c\bar{c}E_{d_{1}}\right)}^{2}\\ & \leq -c_{0}V+D \end{array}$$

where $c_{0}=\min\left(\lambda_{\min}\left(Q-\rho^{2}\left(\lambda_{\max}\left(P^{T}{\mathrm{BB}}^{T}P\right)\right)\right),k_{0}-2,0.5\left(k^{2}-1\right)\right)$ and $D={\left\|\frac{1}{2}{\dot{d}}_{2}^{2}+\frac{1}{2}{\left(c+\bar{c}\right)}^{2}d_{2}^{2}+\frac{1}{2}{\dot{S}}^{2}+\frac{1}{2}{\left(JM\right)}^{2}+{\dot{d}}_{1}^{2}+\frac{1}{2}{\left(c{\bar{E}}_{d_{1}}\right)}^{2}+\frac{1}{2}{\left(c\bar{c}E_{d_{1}}\right)}^{2}\right\|}_{\infty }$ By multiplying $e^{-c_{0}t}$ into (48), it can be obtained as

(49) $$\dot{V}e^{-c_{0}t}\leq -c_{0}Ve^{-c_{0}t}+De^{-c_{0}t}$$

(50) $$\frac{d\left(Ve^{-c_{0}t}\right)}{dt}\leq De^{-c_{0}t}$$

The result of integrating (50) is yielded

(51) $$V(t)\leq V(0)e^{-c_{0}t}+\frac{D}{c_{0}}\left(1-e^{-c_{0}t}\right)$$

Based on References [34,35,36], the proposed control strategy is ultimately uniformly bounded in the presence of matched and mismatched disturbances. The tracking errors and estimation errors of disturbances are bounded. As the Lyapunov function (51), theorem 1 is proved. □

## 4. Simulation Results

### 4.1. System Setup

To examine the practicality of the proposed controller in a bidirectional DC-DC converter, several aspects were considered. First, the bidirectional DC-DC converter was built in the environment of MATLAB/Simulink with components from the Simscape Specialized Power Systems library instead of the numerical dynamic equations (4) to closely reflect its nature. The simulation is conducted with the solver ode4 (Runge-Kutta), and the fundamental sample time is $10^{-6}s$. System parameters are listed in Table 1.

Second, it is assumed in this study that only the inductor current $i_{L}$, the high-side voltage $v_{1}$, and the low-side voltage $v_{2}$ sensing are available to be collected. Meanwhile, other voltage and current sensors are unavailable and only implemented for observation and comparison.

In this section, the simulation results are presented to demonstrate the practicality of the proposed control scheme in voltage regulation of a bidirectional DC-DC converter. Our major objective is not to propose a novel algorithm that would surpass existing algorithms in the literature but rather introduce and study a blend of classical control (SMC) and optimization scheme (ES), thus adding more flexibility to the vast variety of control algorithms targeting disturbance rejection control. For this purpose, comparisons are drawn between the PID control, the constant switching gains, and the proposed adaptive scheme. For the PID control, there are two control loops are implemented including the outer voltage control loop (PI controller 1) and the inner current control loop (PI controller 2). Excepting for switching gains of the adaptive scheme, the parameters of ESO, PID controllers, and SMC are chosen to be the same for all cases, shown in Table 2. Besides, the ES parameters of the proposed adaptive case are recorded in Table 3.

### 4.2. Case Study Results

To verify the effectiveness of the proposed control algorithm on the bidirectional DC-DC converter, three typical study cases including load resistance variations, buck/boost mode switching, and input voltage variation are conducted.

#### 4.2.1. Load Resistance Variations

Two types of resistance variations including a subtle change and two drastic changes are considered to examine the control performance as follows:

$$R_{2}=\{\begin{array}{ll} 100\;\;\Omega & 0.0\;s\leq t<0.1\;s\\ 50\;\;\Omega & 0.1\;s\leq t<0.2\;s\\ 2.5\;\;\Omega & 0.2\;s\leq t<0.3\;s\\ 75\;\;\Omega & 0.3\;s\leq t\leq 0.4\;s \end{array}$$

**Figure 4:**
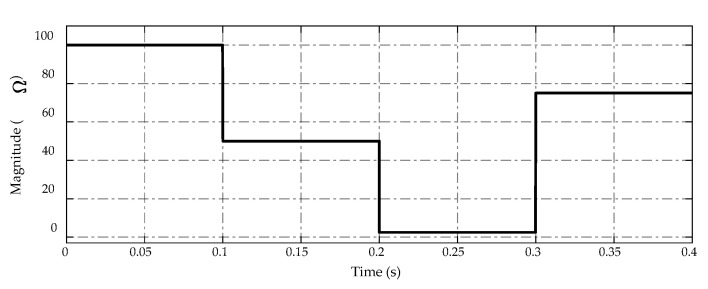
Trajectory of load resistance variations.

With the reference output voltage deliberately kept at $V_{r}=12\;V$, a resistance step from 100 Ω to 50 Ω would create a subtle change in the load current, from $i_{o}=0.12\;\;A$ to $i_{o}=0.24\;\;A$. Meanwhile, resistance steps from 50 Ω to 2.5 Ω and from 2.5 Ω to 75 Ω would create a drastic change, from $i_{o}=0.24\;\;A$ to $i_{o}=4.8\;\;A$, and from $i_{o}=4.8\;\;A$ to $i_{o}=0.16\;\;A$ in the load current. The load resistance variation is shown in Figure 4.

A comparison is drawn between the PID control, the constant switching gains, and the proposed ES-based adaptive scheme. The two constant switching gain candidates were decided based on the highest and lowest switching gains observed in the proposed adaptive scheme case. These gains are selected with η=5450 as the lowest gain and η=9900 as the highest gain and are used under the same testing conditions for comparisons.

**Figure 5:**
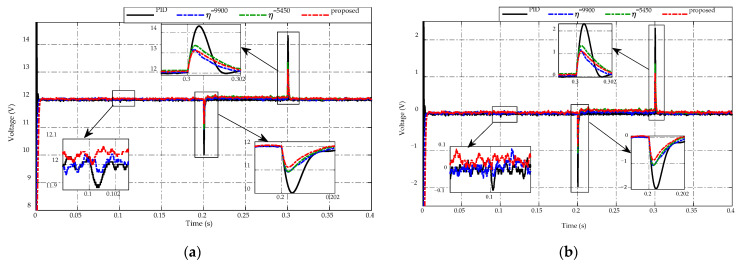
Comparison of output voltage response under load resistance variations. (**a**) Voltage-tracking performance. (**b**) Error effort.

Figure 5 illustrates the voltage-tracking performance of the proposed ES-based adaptive scheme compared to the PID control and the other two constant switching gains. In Figure 5a, the voltage-tracking performances of four strategies are presented, while the corresponding comparative error efforts are described in Figure 5b. These figures are noticeable for all cases at t=0.1  s, the subtle change in load resistance from 100 Ω to 50 Ω causes no abnormal phenomenon in the tracking performance. Meanwhile, at t=0.2  s and t=0.3  s, the effects caused by the drastic changes in load resistance, from 50 Ω to 2.5 Ω with the load current increases 20 times and from 2.5 Ω to 75 Ω with the load current reduces 30 times, are profound. Specifically, at t=0.2  s, the PID control takes a magnitude of peaking phenomenon of approximately 2 V, while two constant switching gains have a smaller peak magnitude of 1 V, and the proposed scheme achieves the lowest voltage peaking with a magnitude of 0.9 V. Similarly, at the transient period of t=0.3  s, the peaking voltage by using PID control reaches to 2.3 V compared to the desired voltage, while the smaller one is 1.2 V from using η=5450 and the best voltage regulation obtains an overshoot voltage of 1 V by the proposed adaptive scheme and η=9900. Such differences indicate that only drastic load resistance variations have a negative impact on the control robustness. As can be seen, different working conditions are likely to require different values of the switching gains that can maintain the system’s performance. Furthermore, the steady-state tracking performance of all control candidates is similar in two intervals t=[0.0  s,  0.2  s]  and t=[0.3  s,  0.4  s] , η=5450 has the worst tracking precision in the middle period t=[0.2  s,  0.3  s] . Besides, control candidates show that adjusting the switching parameter value would not eliminate the peaking phenomena entirely. However, the steady-state tracking accuracy of the proposed control algorithm could reach up to approximately ±0.1 V or 1% the nominal value.

**Figure 6:**
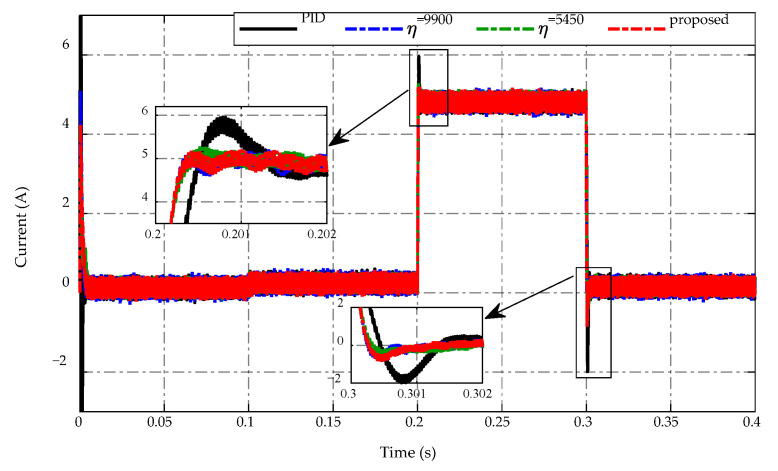
Inductor current response.

The inductor currents of four control strategies are presented in Figure 6. Overall, the proposed strategy improves inductor current performance while reducing current ripple under steady-state working conditions. Meanwhile, when using the PID control, the peaking current value at t=0.2 s is 6 A, which is greater than 1 A in comparison to other strategies. When the load is drastically changed at t=0.3 s, the inductor current adaptation of PID control also performs poorly because the magnitude increases to −2 A, whereas the inductor current of the proposed strategy and two constant switching gains achieves roughly −1 A.

**Figure 7:**
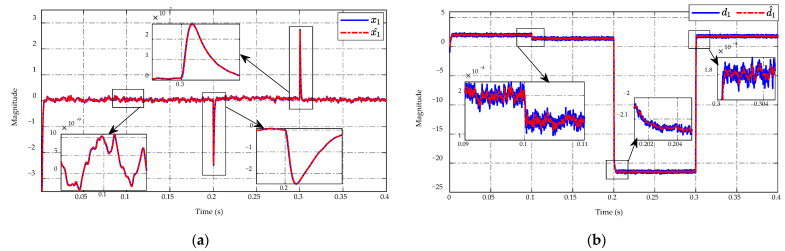
ESO performance in (**a**) state and (**b**) mismatched disturbance estimation.

High precision estimation of the ESO is presented in Figure 7 in which Figure 7a shows the estimated state, while the estimated mismatched disturbance is shown in Figure 7b. Specifically, it can be seen that the estimated state ${\hat{x}}_{1}$ and the estimated mismatched disturbance ${\hat{d}}_{1}$ can track the actual one in a steady-state period and the drastic load resistance changes at t=0.2 s and t=0.3 s. The proposed observer ensures that the asymptotic converges to the small estimation error approximately of $\pm 5\times 10^{-10}$ and $\pm 2\times 10^{-5}$ for state and mismatched disturbance, respectively.

**Figure 8:**
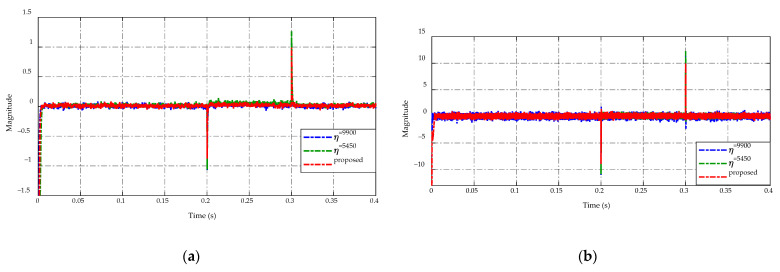
Trajectory of (**a**) prime and (**b**) auxiliary sliding mode variables.

Trajectories of the prime and auxiliary sliding mode variables, σ and *s*, of the proposed controller are described in Figure 8a,b, respectively. The obtained results illustrated that the σ and *s* variables have asymptotic convergence to zero in finite time. Since *s* is partly made of the derivative of σ and σ itself (29), any noise-corrupted signal from system states $x_{1,2}$ and mismatched disturbance estimation ${\hat{d}}_{1}$ could affect σ, as in (28), and eventually get magnified in *s*. The chattering in the trajectory of *s* in Figure 8b has demonstrated the problem and implied that the SMC algorithm is sensitive to noises.

**Figure 9:**
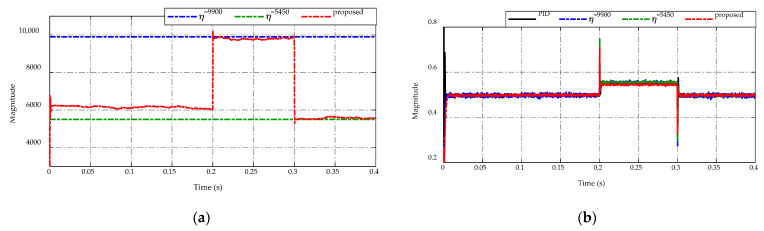
Comparison of (**a**) switching gain adaptation and (**b**) duty cycle command.

Figure 9 displays the comparison of switching gain adaptation in Figure 9a and the duty cycle command in Figure 9b. The implementation of the ES-based adaptation enables adjusting of switching gain η according to the load resistance variations as shown in Figure 9a. This adaptation consequently guarantees the tracking performance and chattering reduction demonstrated in Figure 5 and Figure 6, respectively. In Figure 9b, the duty cycle command of four control strategies is demonstrated. It can be seen that the duty cycles have the same level during t=[0.0 ,  0.2 ] s and t=[0.3 ,  0.4 ] s. When occurring drastic load changes at t=0.2 s and t=0.3 s, the duty cycle of the proposed adaptive strategy has a smaller peaking value than others. In the steady-state period t=[0.2 ,  0.3 ] s, the PID control and two constant switching gains scheme have the same level of duty cycle, while the proposed ES-based adaptive scheme serves a smaller duty cycle value and reduces the chattering effect. Consequently, the flexibility of the proposed adaptive scheme ensures both chattering reductions where possible and tracking precision.

#### 4.2.2. Buck/Boost Mode Switching

**Figure 10:**
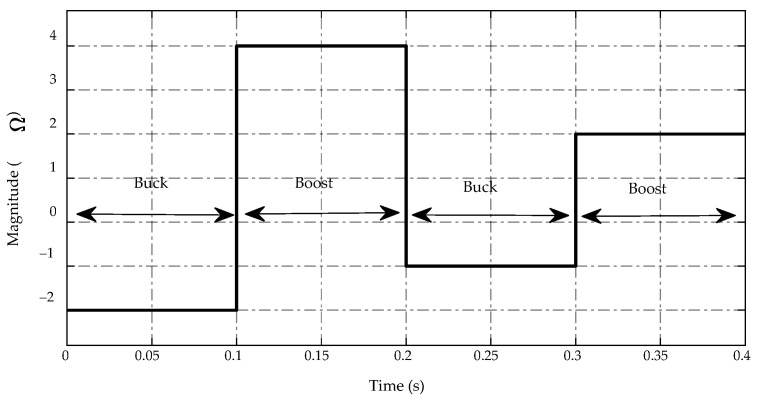
Trajectory of current source demand.

**Figure 11:**
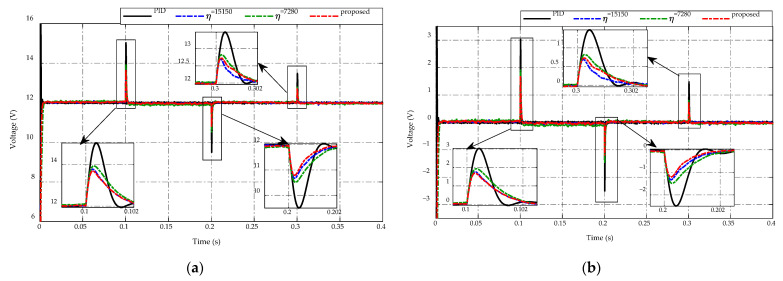
Comparison of low-voltage side response under the buck/boost mode switching. (**a**) Voltage-tracking performance. (**b**) Tracking error.

**Figure 12:**
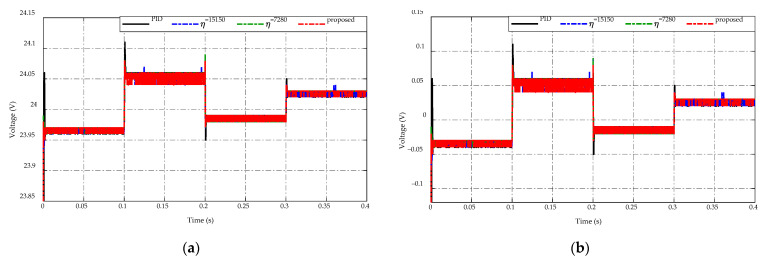
Comparison of high-voltage side response under the buck/boost mode switching. (**a**) Voltage-tracking performance. (**b**) Tracking error.

**Figure 13:**
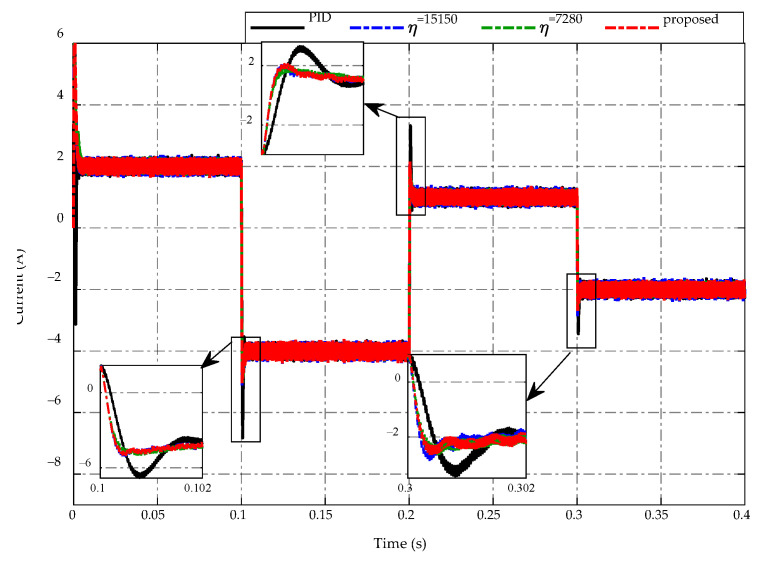
Inductor current response.

**Figure 14:**
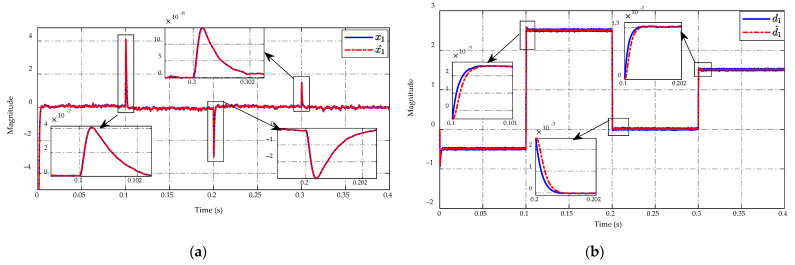
ESO performance in (**a**) state and (**b**) mismatched disturbance estimation.

**Figure 15:**
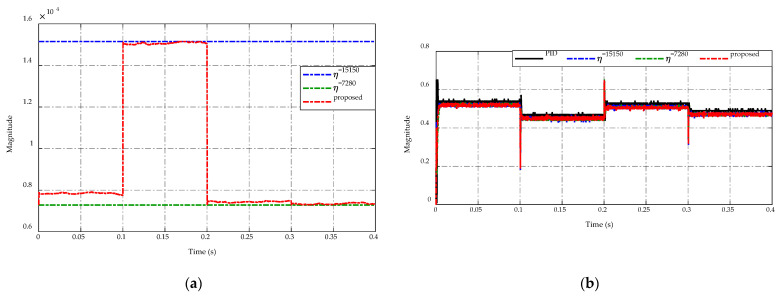
Comparison of (**a**) switching gain adaptation and (**b**) duty cycle command.

In this case study, a controlled current source is used to replace the load resistor on the low-voltage side of a bidirectional DC/DC converter. The current generated by this current source varies between negative and positive values, indicating buck and boost mode, respectively. The main simulation results are presented in Figure 10, Figure 11, Figure 12, Figure 13, Figure 14 and Figure 15, which show similar patterns to the load resistance variations case, especially in mode switching conditions at t=0.1  s, 0.2  s and 0.3  s. The trajectory of the current source demand is described in Figure 10.

In this section, a comparison is conducted to confirm the efficacy of the proposed ES-based adaptive scheme for regulating the high-side and low-side voltages of the converter under the switching conditions of buck and boost mode. The PID control and two constant switching gains are also considered for this comparison. Similarly to the previous section, two constant switching gains are chosen for comparison, with η=7280 serving the lowest value and η=15,150 serving the highest value.

Figure 11 presents the comparison of the low-voltage side response under buck/boost mode switching between the proposed ES-based adaptive scheme compared to the PID control and the other two constant switching gains. The voltage-tracking performances of all strategies are described in Figure 11a, while Figure 11b depicts the corresponding comparative tracking error. It can be seen that the primary target at the low side is to maintain the desired voltage at 12 V. Thus, it is necessary to reduce the voltage chattering and peaking magnitude when switching modes occur to guarantee the voltage quality. Specifically, during the steady-state period of buck and boost mode, the low-voltage side of all control strategies achieves good tracking performance, allowing the obtained voltage to remain constant at 12 V. Meanwhile, the low-voltage side of PID control has the highest peaking magnitude of approximately 3 V when the current source switches from buck mode to boost mode at t=0.1  s with the current change from −2 A to 4 A, while the constant switching gain η=7280 takes a smaller peaking magnitude of 2 V, and the proposed scheme and other constant switching gain η=15,150 achieve the smallest peaking value of 1.8 V. For the working mode changes from boost to buck at t=0.2  s, the proposed adaptive scheme achieves the greatest voltage regulation with the peaking value about −1.2 V, while these peaking magnitudes of other strategies are −1.3 V, −1.5 V, and −2.5 V for the PID control, constant switching gains η=15,150, and η=7280, respectively. Similarly, when switching from buck to boost mode at t=0.3  s with a current change from −1 A to 2 A, the peaking voltage is 1.5 V when using PID control, while the smaller values are 0.8 V and 0.7 V when using η=7280 and proposed adaptive scheme, respectively. The use of adaptive gain η=15,150 achieves the highest voltage response with a peaking magnitude of 0.65 V. The obtained results demonstrate the necessity of adjusting the switching gain in order to decrease peaking phenomena and improve the tracking accuracy for bidirectional DC-DC converter voltage regulation.

For the high-voltage side response, the voltage variations under the buck/boost mode switching of four control strategies are shown in Figure 12 in which Figure 12a depicts the voltage-tracking performances, while Figure 12b presents the corresponding comparative tracking error. Keeping the desired voltage at 24 V is the main requirement on the low side. Overall, a small fluctuation in the range of [−0.05→0.1] V keeps the high-side voltage around the desired value. In particular, when the converter operates in the buck mode during the time t=[0,  0.1]s and t=[0.2,  0.3]s, the battery source will discharge the energy to satisfy load demand, causing a slight degradation of the high-voltage side compared to the desired voltage with the tracking error in the range of [−0.05→0] V as shown in Figure 12b. Meanwhile, the converter works in the boost mode, which controls the charging energy to the battery source, in the interval of t=[0.2,  0.3]s and t=[0.3,  0.4]s. As a result, the high-voltage side fluctuates with the tracking error in the range of [0→0.1] V.

Figure 13 describes the inductor current response by using the proposed adaptive scheme and other strategies. It can be seen that the proposed adaptive strategy achieves a better current ripple than other strategies during the steady-state of buck or boost mode working conditions. Meanwhile, when the converter switches between buck and boost modes at t=0.1 s, 0.2  s, and 0.3 s, the PID control demonstrates the highest peaking current values of −7 A, 3 A, and −3.5 A, respectively. On the other hand, the inductor current of the proposed strategy and two constant switching gains have roughly equal values of −5 A, 2 A, and −3 A at t=0.1 s, 0.2  s, and 0.3 s, respectively.

Figure 14a,b describe the estimated results of the state and mismatched disturbance using the ESO, respectively. In particular, the estimated state ${\hat{x}}_{1}$ shows the high tracking accuracy of the real state $x_{1}$ during the steady-state period and when the converter switches between buck and boost modes at t=0.1 s, 0.2  s, and 0.3 s as shown in Figure 14a. As revealed in Figure 14b, the proposed observer ensures the estimated error of the mismatched disturbance converges to zero under the steady-state working conditions. However, there is still a small estimation error of this mismatched disturbance when mode switching occurs at t=0.1 s, 0.2  s, and 0.3 s as shown in Figure 14b.

The comparison of switching gain adaptation and the duty cycle command is presented in Figure 15a,b, respectively. As shown in Figure 15a, the proposed ES-based adaptation allows for the adjustment of switching gain η to ensure the tracking performance and chattering reduction of the high-side and low-side voltages based on the switching modes of the bidirectional DC/DC converter. As a result, the highest and lowest gains are obtained with η=15,150 and η=7280, respectively. In Figure 15b, the duty cycle command of the PID control and two constant switching gains have the same level during the steady-state working condition. Meanwhile, the proposed strategy produces a smaller duty cycle magnitude than others. When the converter switches the operation modes at t=0.1 s, 0.2  s, and 0.3 s, the duty cycle of four control strategies exhibits the same value of peaking magnitude. Thus, it can be seen that the flexibility of adaptive gains can make sure chattering reductions while also improving the voltage tracking precision.

#### 4.2.3. Input Voltage Variation

**Figure 16:**
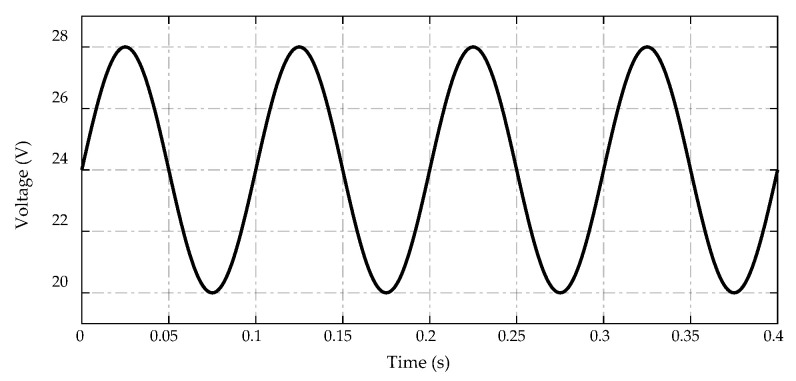
Trajectory of the input voltage.

For this test, a sinusoidal power supply of $V_{S}=24+4\sin(20\pi t)$ is applied to the high-voltage side of the DC/DC converter. The trajectory of this input voltage is described in Figure 16.

**Figure 17:**
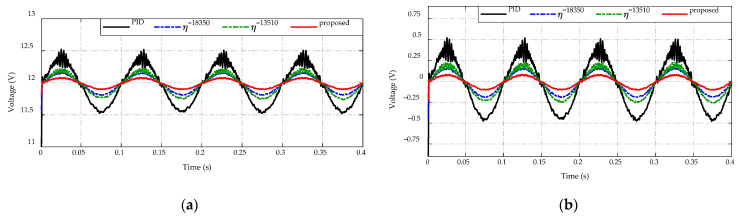
Comparison of output voltage response under the input voltage variation. (**a**) Voltage-tracking performance. (**b**) Error effort.

**Figure 18:**
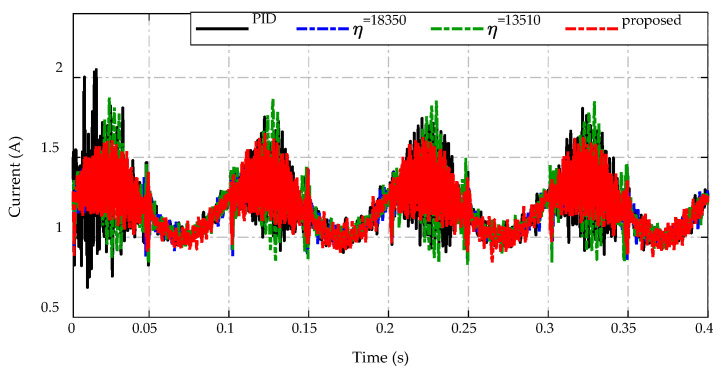
Inductor current response.

**Figure 19:**
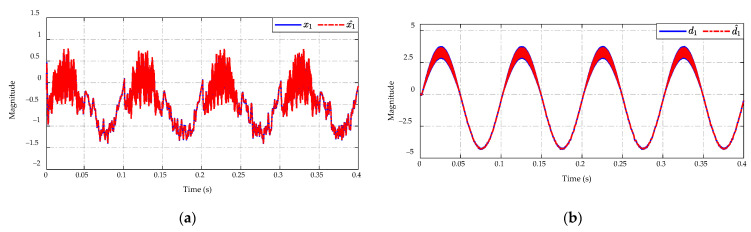
ESO performance in (**a**) state and (**b**) mismatched disturbance estimation.

**Figure 20:**
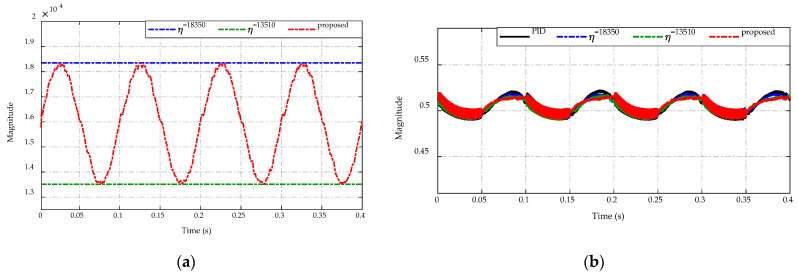
Comparison of (**a**) switching gain adaptation and (**b**) duty cycle command.

In this case study, comparative results are presented to demonstrate the performance of four control strategies under the input voltage variation. In comparison to the proposed control strategy, the PID control and two constant switching gains are used, with η=13,510 being the lowest switching gain and η=18,350 being the highest switching gain. The simulation results of input voltage variation are shown in Figure 17, Figure 18, Figure 19 and Figure 20.

Figure 17 presents the output voltage response of the proposed adaptive scheme, the PID control, and the other two constant switching gains for regulating the output voltage to achieve the desired value at 12 V. It is seen from Figure 17a, the proposed adaptive strategy maintains the output voltage with the least amount of ripple. Meanwhile, constant switching gains show a higher ripple magnitude than the adaptive scheme, and the PID control has the largest output voltage ripple, which affects the output voltage quality of the DC/DC converter. In Figure 17b, the comparative error efforts of four strategies are described. As can be seen, the proposed control strategy achieves the highest tracking accuracy of desired output voltage with the error approximated at ±0.1 V. The PID controller, on the other hand, takes the least tracking accuracy with the error in the range of ±0.5 V, while two constant switching gains obtain greater output voltage adaptation with the tracking error in the range of ±0.2 V and ±0.25 V for η=18,350 and η=13,510, respectively.

The inductor currents of the proposed strategy and others are described in Figure 18. It can be observed that the inductor current shows higher ripple amplitude than other strategies when using the PID controller and constant switching gain η=13,510 with the current fluctuating from 0.7 A to 2 A. Meanwhile, the proposed strategy and constant switching gain η=18,350 achieve the improvement of inductor current ripple in the range of (0.9–1.6) A under conditions of input voltage variation.

The estimation results of the $x_{1}$ state and the mismatched disturbance are shown in Figure 19. The actual values of state and disturbance can be estimated with high precision of estimated state ${\hat{x}}_{1}$ and the estimated mismatched disturbance ${\hat{d}}_{1}$, see Figure 19a,b. The proposed observer can achieve the magnitude of state and mismatched disturbance in the range of $(-1.4\to 0.75)\times 10^{-7}$ and $(-4.4\to 3.7)\times 10^{-3}$, respectively. It can also guarantee that the estimated error converges to zero under the conditions of input voltage variation.

Figure 20a,b present, respectively, the switching gain adaptation for the proposed control strategy and the duty cycle command to control the DC/DC converter. It is seen from Figure 20a, the proposed adaptive scheme can adjust the switching gain η in the range of (13,510–18,350), which ensures the output voltage tracking performance and ripple reduction of the inductor current and the output voltage. For the duty cycle command as described in Figure 20b, the proposed strategy shows a smaller ripple value than others when the sinusoidal input voltage changes in the range of (24–28) V. Besides, it also achieves the narrowest duty cycle magnitude when the input voltage in the range of (20–24) V.

## 5. Conclusions

This paper presented a voltage regulation scheme that combined classical control and optimization for a bidirectional DC-DC converter and it was susceptible to both matched and mismatched disturbances. The theoretical analysis and simulation results explored several pros and cons of the proposed algorithm. First, the control-oriented modeling method captured the dynamics of the converter, allowing different disturbances to be considered and isolated into two common types of disturbances, namely matched and mismatched disturbances. Next, inspired by the work on matched disturbance rejection, an ESO-based CSMC algorithm addressing both mismatched and matched disturbances was proposed, backed by stability analyses, and verified in simulations. With proper parameter selection, the primary objective of tracking control in the presence of disturbance variations and noisy measurement was overall achieved. Nonetheless, the proposed controller was still sensitive to noises and could not completely eliminate the chattering effect. Then, an ES-based adaptive scheme for the switching parameter of SMC was introduced to adapt to different working conditions. The simulated results clearly demonstrated that the proposed strategy performed better than the other three strategies in stabilizing voltage tracking accuracy under the presence of load resistance variations, buck/boost mode switching, and input voltage variation of a bidirectional DC/DC converter. Even though the proposed scheme showed great potential achievements, it could not realize the no-transition performance in voltage controlling the converter, which possessed a fast dynamic response and noise-affected signals. Moreover, the application to voltage regulation of a bidirectional DC-DC converter has revealed the strength and weaknesses of the proposed control algorithm in practice. However, it could ensure tracking precision and chattering reduction in steady-state under different working conditions. Additionally, the presented strategy still has a limitation because verifying the proposed algorithm in actual experiments has not yet been performed. The aforementioned shortcomings open up various possibilities for improvement in both system modeling and control algorithm design in future research such as implementation in a real hardware system, improvement of the adaptive gains response, development of advanced control strategy to system performance, and so on.

## Figures and Tables

**Table 1:** Parameters of the bidirectional DC-DC converter.

Description	Parameter	Value
Battery source	$V_{S}$	24 V
Source internal resistor	$R_{1}$	0.03 Ω
High-side capacitor	$C_{H}$	$200\times 10^{-6}\;\;F$
MOSFET turn-on resistor	$R_{\mathrm{dson}}$	0.01 Ω
Inductor	L	$500\times 10^{-6}\;\;H$
Series resistor	$R_{L}$	0.26 Ω
Low-side capacitor	$C_{L}$	$500\times 10^{-6}\;\;F$
Switching frequency	$f_{switching}$	30 kHz
Reference low-side voltage	$V_{r}$	12 V

**Table 2:** Parameters of the observer and controllers for the comparative study.

Observer/Controllers	Parameter
ESO	$\alpha_{1}=6$ $,\;\alpha_{2}=11$ $,\;\rho =10^{-4}$
PID	$k_{P_{1}}=2$ $,k_{I_{1}}=3000$ $,\;k_{P_{2}}=0.1$ $,k_{I_{2}}=1$
CSMC	c=2500 $,\bar{c}=2000$ $,\;k_{0}=10$

**Table 3:** ES parameters.

Description	Parameter	Value
Objective function (OF) gain	$k_{1}$	0.01
OF error gain	$k_{2}$	$2\times 10^{11}$
OF sliding variable gain	$k_{3}$	4
Forcing frequency	ω	10,125
Demodulation amplitude	*a*	100
Modulation amplitude	*b*	0.05
Learning rate	*k*	226,800
Initial condition	${\hat{\eta }}_{0}$	100

## Data Availability

Not applicable.
